# Exploring General Practitioners' Management of Self‐Harm in Young People: A Qualitative Study

**DOI:** 10.1111/hex.70026

**Published:** 2024-09-09

**Authors:** Faraz Mughal, Benjamin Saunders, Martyn Lewis, Christopher J. Armitage, Lisa Dikomitis, Gillian Lancaster, Ellen Townsend, Carolyn A. Chew‐Graham

**Affiliations:** ^1^ Unit of Academic Primary Care Warwick Medical School, University of Warwick Coventry UK; ^2^ Department of General Practice and Primary Care Melbourne Medical School, University of Melbourne Carlton Victoria Australia; ^3^ School of Medicine Keele University Keele UK; ^4^ Division of Psychology and Mental Health, School of Health Sciences Manchester Centre for Health Psychology, University of Manchester Manchester UK; ^5^ NIHR Greater Manchester Patient Safety Research Collaboration University of Manchester Manchester UK; ^6^ Centre for Health Services Studies and Kent and Medway Medical School University of Kent Kent UK; ^7^ School of Psychology University of Nottingham Nottingham UK

**Keywords:** general practitioner, self‐harm, young people

## Abstract

**Background:**

General practitioners (GPs) are key to the frontline assessment and treatment of young people after self‐harm. Young people value GP‐led self‐harm care, but little is known about how GPs manage young people after self‐harm.

**Aim:**

This study aimed to understand the approaches of GPs to self‐harm in young people and explore their perspectives on ways they might help young people avoid repeat self‐harm.

**Methods:**

We conducted semi‐structured interviews with GPs from the National Health Service in England in 2021. GPs were recruited from four geographically spread clinical research networks and a professional special interest group. Data were analysed using reflexive thematic analysis. The study's patient and public involvement and community of practice groups supported participant recruitment and data analysis.

**Results:**

Fifteen interviews were undertaken with a mean age of participants being 41 years and a breadth of experience in practice ranging from 1 to 22 years. Four themes were generated: GPs' understanding of self‐harm; approaches to managing self‐harm; impact of COVID‐19 on consultations about self‐harm; and ways to avoid future self‐harm.

**Conclusion:**

Negative attitudes towards self‐harm within clinical settings are well documented, but GPs said they took self‐harm seriously, listened to young people, sought specialist support when concerned and described appropriate ways to help young people avoid self‐harm. GPs felt that relationship‐based care is an important element of self‐harm care but feared remote consultations for self‐harm may impede on this. There is a need for brief GP‐led interventions to reduce repeat self‐harm in young people.

**Patient and Public Contribution:**

A study advisory group consisting of young people aged 16–25 years with personal experience of self‐harm and parents and carers of young people who have self‐harmed designed the recruitment poster of this study, informed its topic guide and contributed to its findings.

## 1 Introduction

Self‐harm is defined as intentional self‐injury or poisoning, irrespective of intent, and represents an international public health problem among young people [1]. The lifetime prevalence of self‐harm in 16‐ to 24‐year‐olds in England is reported as 18%, and repeat self‐harm is common, with 15%–25% of adolescents re‐presenting to hospitals for self‐harm within 1 year [2, 3]. The global lifetime prevalence of self‐harm in 12‐ to 18‐year‐olds is 17% [4]. Young people who have self‐harmed are at risk of repeat self‐harm, depression, poorer employment outcomes and suicide [5, 6]. Of young people who have died by suicide, over 50% had previously self‐harmed [7].

Rates of self‐harm in young people documented in general practice electronic health records have increased over the last two decades, and specifically for 13‐ to 16‐year‐old females from March 2020 to March 2022 [6, 8, 9]. Reasons for this observed increase may include rising rates of analgesia and psychotropic medication prescriptions in general practice, greater emergence of common mental health problems in this age group, more frequent help‐seeking, increased social media use, loneliness and more recently disruption and distress from the pandemic [9, 10, 11].

Many young people aged 16–24 years first seek help from their general practitioner (GP) after self‐harm [12]. Young people value GP‐led support for self‐harm and feel care should be personalised [13]. A 2022 narrative review that explored the potential of general practice to support young people after self‐harm identified eight studies but none specifically examined the management practices of GPs for young people [14]. It is known that GPs have a key role in supporting young people after self‐harm but knowledge about how they manage young people is lacking [14, 15]. In addition, there are no effective interventions for GPs to use with young people after self‐harm to prevent future repetition of self‐harm.

There is little evidence on the approaches GPs use with young people who have harmed themselves [14]. The perspectives of GPs on acceptable ways they can support young people can ensure that future interventions are effective, acceptable and scalable. This study aimed to understand the clinical approaches of GPs to self‐harm in young people and explore their perspectives on ways they can support young people to prevent future self‐harm. We also enquired about how COVID‐19 restrictions and changes to consultation mode impacted GPs' approaches.

## 2 Methods

We used a qualitative approach to allow for a rich exploration of clinicians' views to address our aims [16]. Although the study was conceived before COVID‐19, it was conducted early in the pandemic. This study was informed by constructionist epistemology and a critical realist theoretical stance and recognised that individuals have their own subjective insights dependent on their experiences in life [17, 18]. This study is reported according to the Standards for Reporting Qualitative Research [19].

### 2.1 Setting and Participants

This study was conducted in England, and participants were GPs who worked in general practice in the National Health Service (NHS). Trainee or retired GPs, those who only worked outside the NHS and those who worked in non‐routine NHS general practice were not eligible to participate because we wanted to capture the insights of currently practising and qualified GPs, to help improve routine NHS general practice care.

### 2.2 Recruitment

Purposive sampling was used to recruit a diverse sample of GPs based on age, gender, location, years qualified, number of clinical sessions per week and general practice list size. Two recruitment strategies were used. First, four National Institute for Health and Care Research Local Clinical Research Networks (LCRN) were selected around England to gain geographic spread in participant recruitment. The North East and North Cumbria, East of England, West Midlands and South West Peninsula LCRNs agreed to share a recruitment poster with general practices in their areas. Interested eligible clinicians were emailed a participant information pack. Second, F.M. emailed the recruitment poster to the Royal College of General Practitioners Adolescent Health Special Interest Group. Female GPs from the South of England were recruited in response to the demographic characteristics of the first 10 participants to ensure a varied sample of participants. Participants were informed that participation was voluntary and there were no consequences to withdrawing from the study.

### 2.3 Data Collection and Management

**Figure 1:**
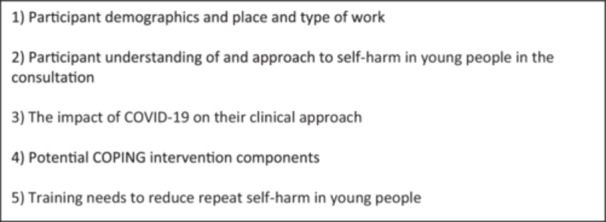
Topics discussed in interviews.

Semi‐structured, in‐depth interviews were used to allow for deep exploration of predetermined interview topics while remaining flexible to the accounts of GPs and allowing for the expansion of unexpected areas of conversation during interviews. A topic guide was used to facilitate data collection during interviews and was iteratively refined as data analysis progressed (see Figure 1 for interview topics). A study risk protocol safeguarded participants if distress was identified during the study.

F.M., who is a GP with experience in qualitative research, conducted all interviews from July to December 2021, remotely via Microsoft Teams or telephone. Interviews were digitally recorded, and recordings were pseudonymised and securely transferred to a professional transcription company for verbatim transcription. Transcripts were checked against audio recordings for accuracy. All participants received a ‘Staying Safe Sheet’ before interviews listing sources of support if needed. Consent was reaffirmed before each interview. Participants were reimbursed £90 for their participation. Data management aligned to Keele University Standard Operation Procedures and adhered to data protection regulations and principles.

Data collection ceased when additional data no longer offered new insights and thus data saturation in this sample of participants was deemed to be achieved [20].

### 2.4 Data Analysis

Interview transcripts were analysed using Braun and Clarke's reflexive thematic analysis that facilitated a rich understanding of GPs' care of young people after self‐harm and supported the generation of themes informed directly from participant data [21]. Interview transcripts were read and reread, and all transcripts were coded at semantic and latent levels, in a collaborative process, with all transcripts being coded independently by at least two authors. Disagreements about codes were resolved through team discussion. Codes were organised into wider categories and initial candidate themes were generated. Candidate themes were refined with patient and public involvement (PPI) and community of practice groups, and higher level recurring themes were agreed on with all authors. Analysis was facilitated by the qualitative software programme NVivo 12 [22].

### 2.5 PPI

Five young people with lived experience of self‐harm, aged between 16 and 25, and three parents or carers of young people who had self‐harmed were recruited to form this study's PPI advisory group. The group, across five remote meetings, helped to design the recruitment poster, refine the interview topic guide and interpret the findings. In meetings, group members discussed and reflected on data extracts openly, which contributed to the generation of candidate themes and the ongoing data analysis.

### 2.6 Reflexivity

F.M. wrote field notes after each interview to support the analysis process. Throughout the study, authors considered how their professional backgrounds in general practice (F.M. and C.C.G.), social science (B.S. and L.D.), clinical trials (M.L. and G.L.), behaviour change (C.J.A.), psychology (E.T. and C.J.A.) and applied health research (B.S., M.L., L.D., C.C.G., G.L., E.T. and C.J.A.) influenced their interpretation of data. Undertaking analysis with researchers from diverse professional backgrounds increased the trustworthiness of the analysis [23].

## 3 Results

Fifteen interviews were conducted, which ranged from 26 to 48 min in length (average: 38 min). All interviews were conducted via Microsoft Teams, but two were transferred to telephone due to poor Internet connection. Participant demographic characteristics are given in Table 1. The mean age of participants was 41 years, and there was the breadth of GP experience in practice ranging from 1 to 22 years. Seven participants were male, and eight, female. GPs worked in practices with registered patient list sizes ranging from 1644 to 39,891. The risk protocol was not activated in this study.

**Table 1:** Participant demographic characteristics.

ID	Age range	Gender	Location	Years qualified	Average number of clinical sessions per week	General practice list size
1	40–49	Male	West Midlands	20	9	3009
2	30–39	Male	NENC	7	6	5348
3	30–39	Male	NENC	1	6	9032
4	40–49	Male	West Midlands	11	10	3627
5	40–49	Female	NENC	15	2	1644
6	40–49	Female	East England	12	3	7432
7	40–49	Male	East England	11	9	14,821
8	40–49	Female	NENC	15	6	3509
9	50–59	Female	East England	22	6	10,836
10	30–39	Male	West Midlands	2	4	7680
11	30–39	Female	London	3	4	16,227
12	40–49	Female	East England	6	2	12,760
13	30–39	Male	South West	2	5	6611
14	40–49	Female	East England	20	6	14,924
15	30–39	Female	South West	8	4	39,891

*Note:* One clinical session = 4 h and 10 min of providing patient care.

Abbreviations: ID, identifier; NENC, North East and North Cumbria.

We generated four themes from the analysis: (1) GPs' understanding of self‐harm; (2) approaches to managing self‐harm; (3) impact of COVID‐19 on consultations about self‐harm; and (4) ways to avoid future self‐harm. We present these below with illustrative data extracts accompanied by a participant identifier.

### 3.1 GPs' Understanding of Self‐Harm

Participants reflected on self‐harm in young people to include not only commonly associated actions such as medication overdose or self‐cutting but also behaviours such as self‐neglect or disordered eating: ‘Commonly thought of things like overdosing medications, cutting … but you could think about other forms of harm in terms of neglecting yourself intentionally …’ (GP2).

Some GPs stated how they felt self‐harm in young people aged 16–25 years sat on a spectrum of severity, ranging from self‐harm thoughts to actual self‐harm episodes, and in the context of episodes, from superficial cutting to attempted hanging. GPs also described how self‐harm presented along with associated mental health problems, and often not as a single problem: ‘We see it often as… part of a larger mental health picture… mostly a depression and anxiety picture’ (GP15).

Some participants felt that self‐harm was motivated by seeking emotional release in young people, whereas others explained how they felt self‐harm was a cry for help when a young person was experiencing a crisis, but all GPs recognised self‐harm as a serious problem needing clinical attention: ‘I think no matter how you look at it … it's something that is certainly serious and … can't be ignored’ (GP1).

Most GPs stated that young people generally self‐harmed without the intent of suicide but did acknowledge that this was not always the case: ‘I appreciate that they're very closely linked, but yes absolutely distinct, so I understand people feeling a drive or a need to harm themselves as a release … but not necessarily wanting that to end their lives. But equally there will be a cohort for whom that will be a precursor to that’ (GP14).

### 3.2 Approaches to Managing Self‐Harm

Most participants described taking time to gather a thorough clinical history, including why and how self‐harm occurred, to inform their management plan: ‘I mean I'd be doing an assessment, so normally I'd be finding out … what's going on at home, school, college, job … you're trying to work out why they're kind of doing it, what they're doing … and then trying to make some kind of plan moving forward’ (GP8). Clinicians talked about conducting a process of assessment, including risk categorisation, for the risk of future self‐harm or suicide to support their clinical decision‐making: ‘I think risk stratification as well in terms of how likely they are to go on to do this again. How could the behaviours escalate? Is there a risk of suicide … And from there, risk management … the ultimate thing is … I suppose a threshold for a referral in terms of is this something that's suitable to be managed in primary care’ (GP3).

Some GPs explained they would enquire about any co‐existing mental health problems such as anxiety or low mood, and if detected, would offer treatment. Other GPs said they focused on addressing any psychological pain to help the young person avoid future self‐harm: ‘I think I lean [towards], you know, the underlying distress, trying to improve that’ (GP9). Participants also described attending to the physical consequences of the self‐harm, such as inspecting skin wounds after self‐cutting to see if there is an infection or if a dressing is needed: ‘If they said “oh, I've just cut my skin with a sharp,” and then I'll say “you know is it okay if I can have a look at that?, and it's important that we know it's not going to get infected”’ (GP14).

Most clinicians reported being proactive in their follow‐up of young people after self‐harm: ‘I generally will always follow up a young person, normally fairly quickly … within a week or two … to see how things evolve and to see whether they're actually you know, taking any action’ (GP10). Many participants commented on ensuring that the young person is safe after the consultation, which included educating the young person on when to seek help and providing crisis care information.

Participants explained how they guided young people to counselling and well‐being services after self‐harm. Other participants described referring young people to Child and Adolescent Psychiatry services for specialist support following self‐harm: ‘It might be sort of family issues that are leading to self‐harm in which case sometimes a referral to Child and Adolescent Mental Health Services’ (GP5); but one GP stated how accessing specialist services for young people after self‐harm was challenging because of high referral thresholds, and explained how they need to manage young people at high risk without specialist input. Some GPs described referring young people to emergency mental health teams such as crisis teams if they were worried about escalating self‐harm behaviour or immediate risk of suicide: ‘The next step would be, have they got any intention to kill themself … if I have a doubt… then it would be crisis team involvement’ (GP4).

Participants explained how they attempted to listen and be non‐judgemental in consultations: ‘I do always want to try and create, you know, that open, non‐judgemental consultation style as best as possible’ (GP10). Clinicians also described acknowledging and validating the self‐harm in young people. Some GPs talked about how a positive rapport with a patient can lead to shared ownership in addressing self‐harm: ‘I think, you know, the person needs to not feel talked to, so it's really important that you get some kind of shared ownership of what you're trying to reduce’ (GP9).

### 3.3 Impact of COVID‐19 on Consultations About Self‐Harm

Participants reflected on how moving to remote (telephone, video, email or online) consultations to mitigate the risks of COVID‐19 contagion negatively impacted their ability to deliver relationship‐based care (an enhanced level of care consisting of GP continuity and trust, mutual respect and sharing of power) [24]: ‘A lot of the consultations were done over the phone because of COVID … I think that's hugely impacted our ability to deal with it. I think the importance of connection, and rapport, and somebody seeing your face’ (GP12). GPs described the challenge of managing self‐harm during remote consultations: ‘When you see them remotely [telephone consults] you don't get any of the cues because you're not getting any of the visual clues … you know how you read people, that's one of my strengths … you can't do it over the phone’ (GP1).

Although most GPs perceived remote consultations to negatively affect self‐harm management, some felt that more young people might access a GP following self‐harm: ‘I think COVID obviously, you know, the way it's changed our practice in terms of remote consultations I think that suits young people better, I think it's easier for them to access us that way’ (GP10). Other clinicians felt that COVID‐19 restrictions led to fewer patients seeking GP care after self‐harm because of public health messaging during COVID‐19 about the strain on the NHS services.

One participant described the ability to better prepare for a telephone self‐harm consultation after receiving an online consultation request about self‐harm thoughts: ‘If we do get an e‐consult saying, “we've got this and am having these thoughts”, we can ring them back … it's not going to be a five‐minute conversation. It's going to be a 15, 20, 30 minutes and you can also have all the kind of guides … you can have them preloaded and you can text them these things’ (GP3).

### 3.4 Ways to Avoid Future Self‐Harm

Most GPs stated that advising patients about specific distraction techniques such as holding ice cubes or using distress tolerance methods may help them avoid repeat self‐harm: ‘A clear strategy for managing the distress in the moment. So for me I used the [name of app] … it might be a breathing technique … appreciating that some people might need comfort and some people might need an outlet’ (GP14).

Clinicians commented on how providing information to young people, as well as setting goals, may help to reduce future self‐harm: ‘You can help them formulate those goals and setting small goals like that, sort of homework’ (GP14). Some participants felt that signposting patients to supporting services, including seeking input and expertise from general practice colleagues, may help them avoid self‐harm: ‘I would have mental health workers within the practice if I could. I think in terms of specifically around self‐harm … “lets, you know get you [patient] in now, come down and have a chat, I've got somebody next door [mental health worker], and we'll have a chat about it”’ (GP8).

Other GPs described the importance of having a consistent approach to providing care for young people after self‐harm: ‘This is why I really like the idea of having a sort of standardised approach … Because at the moment we'll say, “Go to the website for some online counselling”, but I think a lot of the time they don't go on it’ (GP11).

Some participants stated that encouraging patients to have greater insight, self‐empowerment to attempt self‐care options and self‐enablement may support them in developing skills to avoid further self‐harm: ‘If it was generally mild self‐harm, then I would find out what they felt they could do to support themselves first of all and then signpost them to resources that might be helpful’ (GP12) and ‘because ultimately it's got to be something that comes from them’ (GP6).

## 4 Discussion

This study is the first to describe GP perspectives on their approaches to managing young people after self‐harm, how COVID‐19 impacted self‐harm consultations and ways GPs could help young people avoid future self‐harm. Clinicians understood self‐harm to be broad in types of behaviours, associated with mental health problems, driven by underlying psychological distress and varying in severity. GPs reported taking time to understand the context for self‐harm, identifying and treating co‐existing mental illness, proactively following young people, referring them to supporting services, and acknowledging the self‐harm. COVID‐19 restrictions led to GPs consulting remotely in practice, including with young people about self‐harm. Distraction techniques, information‐giving and encouraging self‐empowerment were thought to be appropriate ways for GPs to help young people refrain from future self‐harm.

### 4.1 Comparison With Existing Literature

GPs from Scotland and England have previously described self‐harm in young people as mostly involving self‐cutting and self‐poisoning [25, 26]. We found that GPs understood self‐harm to be broader than these methods and included self‐neglect. In this study, GPs identified self‐harm to be due to psychological distress and as a way of seeking help in crises. We have identified the functions of self‐harm in 16‐ to 25‐year‐olds in a recent qualitative study; the function of self‐harm as ‘handling emotional states’ would incorporate self‐harm as a result of underlying distress [13]. GPs have also recognised self‐harm as a coping tool for young people [27]. A recent systematic review highlighted ongoing negative attitudes in clinical services towards self‐harm management, including patients feeling that assessments were rushed and superficial and that staff did not listen to their experiences [28]. However, we identified that GPs attempted to build rapport with young people, taking time to listen and understand the self‐harm context and identifying any co‐existing mental health illness.

Australian GPs have explained using assessment tools to predict the future risk of self‐harm or suicide because these tools serve as useful prompts and help them differentiate levels of risk; however, they had concerns about their predictive ability and their impact on the therapeutic relationship [29]. GPs in the present study reported using risk assessment approaches that included risk stratification to inform clinical decisions. The National Institute for Health and Care Excellence (NICE) 2022 self‐harm guideline states that global risk stratification into low, medium or high‐risk categories should not inform treatment decisions because of the poor ability of such approaches to predict future self‐harm and suicide in individuals [30].

Observational data from early COVID‐19 (from March to June 2020) found that young people were more likely (rate ratio 2.43, 95% CI 2.33–2.54) to have had a remote consultation within 3 months after a recorded self‐harm episode compared to the pre‐pandemic period, strengthening the accounts we uncovered about the adoption of remote consultations in early COVID‐19 [31]. GPs have previously described feelings of powerlessness at not being able to do more for young people after self‐harm [29]. Conversely, young people who have self‐harmed have said that self‐help resources can help them manage their self‐harm and can provide conversation prompts for GPs to use in practice [26].

## 5 Strengths and Limitations

The diversity of participants in terms of age, gender, clinical experience and work location is a strength. The analysis approach facilitated a recursive and iterative process, grounding themes closely to the original data [21]. The involvement of the PPI and community of practice groups in the interpretation of data increased the credibility of our results. We adapted the data collection method and used remote interviews to deliver the study during COVID‐19. These findings are likely applicable to family medicine doctor‐led self‐harm care internationally; however, this transferability may be limited by changing socioeconomic contexts globally.

There were some limitations. The GPs who consented to the interview were likely interested in young people's mental health; thus, views expressed may not be representative of all GPs in England. There was also the possibility of participants only disclosing partial accounts in some areas of interviews because the interviewer, F.M., is also a GP, and so participants may have felt that their clinical practice was being assessed [32].

## 6 Implications for Practice and Future Research

GPs need to recognise that self‐harm in 16‐ to 25‐year‐olds can serve different functions but should be taken seriously. GPs can attempt to undertake a risk formulation which is a collaborative process that can occur across consultations summarising immediate risks, why they are occurring, and protective factors, and in turn tailor treatment to unmet clinical needs [33]. GPs have suggested some ways that may help young people who have self‐harmed, but these require rigorous evaluation. Relationship‐based care is an important element of self‐harm management, and teams can attempt to facilitate this by organising continuity of GP care [34].

With remote consulting likely to be a core future consultation mode in general practice, GPs should recognise that although remote consultations for self‐harm care may expand the reach of support, they may lead to the missing of non‐verbal cues and hinder rapport building, thus impacting on the development of relationship‐based care [35]. Well‐resourced and adequately evaluated self‐harm services in primary care would support clinicians where waiting lists for specialist care are long.

Future research should explore how remote GP consulting influences self‐harm care, including capturing GP and young people ethnicity data, and how parents and carers are involved in the care of young people. GPs require effective strategies and treatments to use with young people after self‐harm; thus, there is a need for the development and testing of new GP‐led brief interventions for young people to reduce repeat self‐harm.

## Author Contributions

**Faraz Mughal:** conceptualisation, funding acquisition, investigation, methodology, writing–review and editing, writing–original draft, formal analysis, project administration, data curation. **Benjamin Saunders:** supervision, writing–review and editing, formal analysis. **Martyn Lewis:** writing–review and editing, formal analysis, supervision. **Christopher J. Armitage:** supervision, formal analysis, writing–review and editing. **Lisa Dikomitis:** writing–review and editing, supervision, formal analysis. **Gillian Lancaster:** writing–review and editing, supervision, formal analysis. **Ellen Townsend:** supervision, writing–review and editing, formal analysis. **Carolyn A. Chew‐Graham:** writing–review & editing, supervision, formal analysis.

## Ethics Statement

This study was granted ethical approval from Keele University Faculty of Medicine and Health Sciences Research Ethics Committee (MH‐210180) and NHS Health Research Authority approval (IRAS: 294180).

## Conflicts of Interest

F.M. was a member of the 2022 NICE Self‐Harm Guideline [NG225] development committee. The other authors declare no conflicts of interest.

## Data Availability

Reasonable data requests will be considered by the lead author.

## References

[1] D. Knipe , P. Padmanathan , G. Newton‐Howes , L. F. Chan , and N. Kapur , “Suicide and Self‐Harm,” Lancet 399, no. 10338 (May 2022): 1903–1916, 10.1016/s0140-6736(22)00173-8. 35512727

[2] S. McManus , D. Gunnell , C. Cooper , et al., “Prevalence of Non‐Suicidal Self‐Harm and Service Contact in England, 2000‐14: Repeated Cross‐Sectional Surveys of the General Population,” Lancet Psychiatry 6, no. 7 (June 2019): 573–581, 10.1016/s2215-0366(19)30188-9. 31175059 PMC7646286

[3] K. Hawton and L. Harriss , “Deliberate Self‐Harm by Under‐15‐Year‐Olds: Characteristics, Trends and Outcome,” Journal of Child Psychology and Psychiatry 49, no. 4 (April 2008): 441–448, 10.1111/j.1469-7610.2007.01852.x. 18081755

[4] D. Gillies , M. A. Christou , A. C. Dixon , et al., “Prevalence and Characteristics of Self‐Harm in Adolescents: Meta‐Analyses of Community‐Based Studies 1990‐2015,” Journal of the American Academy of Child & Adolescent Psychiatry 57, no. 10 (October 2018): 733–741, 10.1016/j.jaac.2018.06.018. 30274648

[5] B. Mars , J. Heron , C. Crane , et al., “Clinical and Social Outcomes of Adolescent Self Harm: Population Based Birth Cohort Study,” BMJ (Clinical Research Ed.) 349 (October 2014): 5954, 10.1136/bmj.g5954. PMC4205277 25335825

[6] C. Morgan , R. T. Webb , M. J. Carr , et al., “Incidence, Clinical Management, and Mortality Risk Following Self Harm Among Children and Adolescents: Cohort Study in Primary Care,” BMJ (Clinical Research Ed.) 359 (October 2017): j4351, 10.1136/bmj.j4351. PMC5641980 29046278

[7] “Suicide by Children and Young People,” National Confidential Inquiry into Suicide and Homicide by People with Mental Illness (NCISH), 2017, http://research.bmh.manchester.ac.uk/cmhs/research/centreforsuicideprevention/nci/reports/cyp_2017_report.pdf.

[8] M. J. Carr , D. M. Ashcroft , E. Kontopantelis , et al., “The Epidemiology of Self‐Harm in a UK‐Wide Primary Care Patient Cohort, 2001–2013,” BMC Psychiatry 16, no. 1 (2016): 53, 10.1186/s12888-016-0753-5. 26923884 PMC4770684

[9] A. M. Trafford , M. J. Carr , D. M. Ashcroft , et al., “Temporal Trends in Eating Disorder and Self‐Harm Incidence Rates Among Adolescents and Young Adults in the UK in the 2 Years Since Onset of the COVID‐19 Pandemic: A Population‐Based Study,” Lancet Child & Adolescent Health 7, no. 8 (August 2023): 544–554, 10.1016/s2352-4642(23)00126-8. 37352883

[10] F. Mughal , A. House , N. Kapur , R. T. Webb , and C. A. Chew‐Graham , “Suicide Prevention and COVID‐19: The Role of Primary Care During the Pandemic and Beyond,” British Journal of General Practice 71, no. 706 (May 2021): 200–201, 10.3399/bjgp21X715637. PMC8087298 33926869

[11] E. G. Tyrrell , D. Kendrick , K. Sayal , and E. Orton , “Poisoning Substances Taken by Young People: A Population‐Based Cohort Study,” British Journal of General Practice 68, no. 675 (October 2018): e703–e710, 10.3399/bjgp18X698897. PMC6145981 30201829

[12] A. Marchant , S. Turner , L. Balbuena , et al., “Self‐Harm Presentation Across Healthcare Settings by Sex in Young People: An E‐Cohort Study Using Routinely Collected Linked Healthcare Data in Wales, UK,” Archives of Disease in Childhood 105, no. 4 (April 2020): 347–354, 10.1136/archdischild-2019-317248. PMC7146921 31611193

[13] F. Mughal , C. A. Chew‐Graham , O. O. Babatunde , B. Saunders , A. Meki , and L. Dikomitis , “The Functions of Self‐Harm in Young People and Their Perspectives About Future General Practitioner‐Led Care: A Qualitative Study,” Health Expectations 26, no. 3 (June 2023): 1180–1188, 10.1111/hex.13733. 36797811 PMC10154897

[14] F. Mughal , L. Dikomitis , O. O. Babatunde , and C. A. Chew‐Graham , “The Potential of General Practice to Support Young People Who Self‐Harm: A Narrative Review,” BJGP Open 6, no. 1 (March 2022): BJGPO.2021.0159, 10.3399/bjgpo.2021.0159. 35135818 PMC8958734

[15] F. Mughal , M. I. Troya , L. Dikomitis , C. A. Chew‐Graham , N. Corp , and O. O. Babatunde , “Role of the GP in the Management of Patients With Self‐Harm Behaviour: A Systematic Review,” British Journal of General Practice 70, no. 694 (2020): e364–e373, 10.3399/bjgp20X708257. PMC7015161 32041771

[16] J. Creswell , Qualitative Inquiry and Research Design: Choosing Among Five Traditions (California: SAGE Publications Inc, 1997).

[17] M. Crotty , The Foundations of Social Research: Meaning and Perspective in the Research Process (London: SAGE Publications Ltd, 1998).

[18] R. Bhaskar , A Realist Theory of Science, 2nd illustrated ed. (Hertfordshire: Harvester Wheatsheaf, 1978).

[19] B. C. O'Brien , I. B. Harris , T. J. Beckman , D. A. Reed , and D. A. Cook , “Standards for Reporting Qualitative Research: A Synthesis of Recommendations,” Academic Medicine 89, no. 9 (2014): 1245–1251. 24979285 10.1097/ACM.0000000000000388

[20] B. Saunders , J. Sim , T. Kingstone , et al., “Saturation in Qualitative Research: Exploring Its Conceptualization and Operationalization,” Quality & Quantity 52, no. 4 (2018): 1893–1907, 10.1007/s11135-017-0574-8. 29937585 PMC5993836

[21] V. Braun and V. Clarke , Thematic Analysis: A Practical Guide (London: SAGE Publications Ltd, 2022).

[22] QSR International Pty Ltd, *NVivo (Version 12)* (Denver, CO, USA: QSR International Pty Ltd, 2018).

[23] K. L. Henwood and N. F. Pidgeon , “Qualitative Research and Psychological Theorizing,” British Journal of Psychology 83 (February 1992): 97–111. 1559146 10.1111/j.2044-8295.1992.tb02426.x

[24] R. Jones , “General Practice in the Years Ahead: Relationships Will Matter More Than Ever,” British Journal of General Practice 71 (2021): 4–5. 10.3399/bjgp21X714341 PMC7759379 33372084

[25] A. Chandler , C. King , C. Burton , and S. Platt , “The Social Life of Self‐Harm in General Practice,” Social Theory & Health: STH 18, no. 3 (September 2020): 240–256, 10.1057/s41285-020-00139-9. 32855622 PMC7115977

[26] D. Bailey , L. Kemp , N. Wright , and G. Mutale , “Talk About Self‐Harm (TASH): Participatory Action Research With Young People, GPs and Practice Nurses to Explore How the Experiences of Young People Who Self‐Harm Could Be Improved in GP Surgeries,” Family Practice 36, no. 5 (October 2019): 621–626, 10.1093/fampra/cmz006. 30796781

[27] F. Fox , P. Stallard , and G. Cooney , “GPs Role Identifying Young People Who Self‐Harm: A Mixed Methods Study,” Family Practice 32, no. 4 (2015): 415–419, 10.1093/fampra/cmv031. 25957173 PMC4507514

[28] T. Uddin , A. Pitman , G. Benson , Z. Kamal , K. Hawton , and S. Rowe , “Attitudes Toward and Experiences of Clinical and Non‐Clinical Services Among Individuals Who Self‐Harm or Attempt Suicide: A Systematic Review,” Psychological Medicine 54, no. 1 (January 2024): 13–31, 10.1017/s0033291723002805. 37772412

[29] I. Bellairs‐Walsh , S. J. Byrne , S. Bendall , et al., “Working With Young People at Risk of Suicidal Behaviour and Self‐Harm: A Qualitative Study of Australian General Practitioners' Perspectives,” International Journal of Environmental Research and Public Health 18, no. 24 (December 2021): 12926, 10.3390/ijerph182412926. 34948536 PMC8701929

[30] “Self‐Harm: Assessment, Management and Preventing Recurrence [NG225],” National Institute for Health and Care Excellence, 2022, https://www.nice.org.uk/guidance/ng225. 36595613

[31] S. Steeg , M. Carr , L. Trefan , et al., “Primary Care Clinical Management Following Self‐Harm During the First Wave of COVID‐19 in the UK: Population‐Based Cohort Study,” BMJ Open 12, no. 2 (February 2022): e052613, 10.1136/bmjopen-2021-052613. PMC8844953 35165109

[32] C. A. Chew‐Graham , “Qualitative Research and the Problem of Judgement: Lessons From Interviewing Fellow Professionals,” Family Practice 19, no. 3 (June 2002): 285–289, 10.1093/fampra/19.3.285. 11978720

[33] F. Mughal , L. Clarke , R. Connolly , A. Y. T. Lee , L. Quinlivan , and N. Kapur , “Improving the Management of Self‐Harm in Primary Care,” British Journal of General Practice 73 (2023): 148–149. 10.3399/bjgp23X732297 PMC10049611 36997200

[34] F. Mughal , L. Dikomitis , O. O. Babatunde , and C. A. Chew‐Graham , “Experiences of General Practice Care for Self‐Harm: A Qualitative Study of Young People's Perspectives,” British Journal of General Practice 71 (May 2021): e744–e752, 10.3399/bjgp.2021.0091. PMC8340729 33950851

[35] F. Mughal , H. Atherton , H. Awan , et al., “The Impact of Remote Consultations on Brief Conversations in General Practice,” BJGP Open 6, no. 2 (June 2022): BJGPO.2021.0199, 10.3399/bjgpo.2021.0199. 35217511 PMC9447317

